# A pilot study of multi-modal pain management for same-day discharge after minimally invasive repair of pectus excavatum (Nuss procedure) in children

**DOI:** 10.1007/s00383-023-05429-7

**Published:** 2023-03-26

**Authors:** Sophia Akinboro, Rebecca John, Troy Reyna, Rachel Davis, Christine Ayoub, Rebecca Sangster, Joseph Kim, Hai Nguyen, Claudia Moreno, Yigit Guner, Laura Goodman, Peter T. Yu, Tricia Morphew, Mustafa Kabeer

**Affiliations:** Children’s Hospital of Orange County, Orange, USA

**Keywords:** Pectus, Cryoablation, Nuss, Paravertebral block, Pain

## Abstract

**Background:**

Despite advancements in minimally invasive repair of pectus excavatum (MIRPE), Nuss procedure, postoperative pain control remains challenging. This report covers a multimodal regimen using bilateral single-shot paravertebral block (PVB) and bilateral thoracoscopic intercostal nerve (T3–T7) cryoablation, leading to significant reduction in length of stay (LOS) and high rate of same-day discharge.

**Methods:**

This is a comparative study of pain management protocols for patients undergoing the Nuss procedure at a single center from 2016 through 2020. All patients underwent the the same surgical technique for the treatment of pectus excavatum at a single center. Patients received bilateral PVB with continuous infusion (Group 1, *n* = 12), bilateral PVB with infusion and right-side cryoablation (Group 2, *n* = 9), or bilateral single-shot PVB and bilateral cryoablation (Group 3, *n* = 17). The primary outcome was LOS with focus on same-day discharge, and the secondary outcome was decreased opioid usage.

**Results:**

Eleven of 17 patients in Group 3 (65%) (bilateral single-shot PVB and bilateral cryoablation) were discharged the same day as surgery. The remaining Group 3 patients were discharged the following day with no complications or interventions. Compared to Group 1 (no cryoablation), Group 3 had shorter LOS (median 4.4 days vs. 0.7 days, respectively, p < 0.001) and significantly decreased median opioid use on the day of surgery (0.92 mg/kg vs. 0.47 mg/kg, *p* = 0.006).

**Conclusion:**

Findings demonstrate the feasibility of multimodal pain management for same-day discharge after the Nuss procedure. Future multisite studies are needed to investigate the superiority of this approach to established methods.

**Level of Evidence:**

III.

**Supplementary Information:**

The online version contains supplementary material available at 10.1007/s00383-023-05429-7.

## Introduction

The developmental chest wall deformity known as pectus excavatum causes inward displacement of the sternum and adjacent costal cartilage [1]. Pectus excavatum affects approximately 1 in 400 live births, with a fourfold increase in risk among males [1]. Most cases of pectus excavatum are first observed during adolescence, with worsening during the rapid growth associated with puberty. Affected patients may have restricted breathing and exercise intolerance due to right heart compression, along with psychosocial anxiety [2, 3]. Historically, surgical correction of pectus excavatum was achieved using the Ravitch procedure for exposure and dissection of the bony anterior chest wall, resection of the costal cartilage involved, and fracture and displacement of the sternum. In 1998, Dr. Donald Nuss reported a technique for minimally invasive repair of pectus excavatum that has largely supplanted the Ravitch procedure as the standard approach to treatment. The Nuss procedure is based on use of a retrosternal metallic bar to reshape the thorax over the course of 2–3 years before removal [4, 5]. However, despite advantages over the Ravitch procedure which include minimal intraoperative blood loss, shorter duration of surgery, earlier return to full activity, and overall improvement in quality of life, the Nuss procedure requires active stretching of the chest wall and intercostal nerves, which have been associated with severe and prolonged postoperative pain [5–10].

Opioids are widely used to control pain in patients who have undergone the Nuss procedure, and the side effects associated with opioid use may be severe, ranging from itching, sedation, nausea, and constipation to respiratory depression. Furthermore, the habitual use of opioids can lead to dependence. Therefore, decreasing opioid usage can provide short-term as well as long-term benefits for patient safety and quality of life. The current epidemic of opioid misuse and abuse has given urgency to move towards effective opioid-conscious protocols [13, 15]. The standardization of perioperative care for enhanced recovery after surgery (ERAS), which encourages opioid-sparing analgesia and improves surgical outcomes after many surgical procedures [11–14] including the Nuss procedure. With this goal in mind, pediatric surgeons at our institution sought to establish a standardized approach to postoperative pain management for Nuss procedure patients. Randomized clinical trials and retrospective studies have shown that cryoablation provides long-acting regional nerve blockade that outlasts injections and catheter-based delivery systems, providing analgesia throughout the postoperative period when pain is most severe [16–20]. After cryoablation, sensation returns as the nerve axons regenerate within the intact nerve sheath [16, 21]. Cryoanalgesia is particularly ideal for patients undergoing the Nuss procedure because the intercostal nerves are directly visualized thoracoscopically and easily ablated [22].

The increasingly widespread use of cryoablation combined with the desire to reduce opioid use led us to establish a multi-modal pain management protocol. The purpose of this study was to assess if this protocol utilizing procedural based analgesia could improve pain management and lead to same-day discharge after the Nuss procedure. Therefore, in this comparative pilot study of Nuss procedure patients, we hypothesized that the use of bilateral single-shot paravertebral block (PVB) with bilateral cryoablation and an oral medication regimen would result in reduced length of stay (LOS) and decreased overall opioid usage when compared to right-side cryoablation and bilateral paravertebral block with continuous infusion catheter (PVBc) and compared to bilateral PVBc alone (no cryoablation).

## Materials and methods

### Study design

This was a comparative study of pain management protocols for patients undergoing the Nuss procedure at a single center during the period from 2016 through 2020. The study was approved by the institutional review board of Children’s Hospital of Orange County (IRB #1,911,147). All patients underwent the Nuss procedure with the same surgical technique for the treatment of pectus excavatum. Data collected for study inclusion included demographics, Haller index, presenting symptoms. All patients included in the study participated in a mentorship program where each patient had a chance to connect with other patients and families who had recently undergone the Nuss procedure at our institution. All participants underwent full pulmonary and cardiology evaluations (including cardiac magnetic resonance imaging [MRI]) preoperatively.

Groups were established by differentiating between the pain management protocol each patient received while inpatient following scheduled surgery. A total of 38 patients were included based on the inclusion criteria, however two groups were retrospective with chart review only. Group 1 (*n* = 12) was a retrospective cohort, with bilateral PVB with placement of PVB-catheter (PVBc) for continuous analgesic infusion and no cryoablation; Group 2 (*n* = 9), also retrospective, had combined bilateral PVB with PVBc and right-side thoracoscopic cryoablation only. During this time period, only single side cryoablation was performed given the paucity of information regarding the safety of cryoablation specifically as it related to recovery of sensation and concerns over lifelong chest wall numbness. Since that time, there was a study that reported complete recovery of sensation in pediatric patients under 21 years of age [23]. A new multi-modal pain management protocol was established for all postoperative Nuss patients regardless of inclusion in this study. Thus, group 3 (*n* = 17) was prospective using this new protocol and consisted of patients receiving bilateral single-shot PVB (no catheter) combined with bilateral thoracoscopic cryoablation and a set pre- and post-operative protocolized medication regimen. Groups were similar in terms of age and weight (ANOVA, *p* = 0.827 and *p* = 0.682, respectively) (Table 1). However, the proportion of males was significantly lower in Group 2 (right-side cryoablation), compared to Groups 1 and 3 (66.7 vs. 94.1% and 100%, respectively, *p* = 0.034).

**Table 1:** Description of study population by treatment group

	Overall *N* = 38	Treatment group			*P* value^€^
		Group 1 (Pre-cryo) *N* = 12	Group 2 (Cryo-right) *N* = 9	Group 3 (Cryo-bilateral) *N* = 17	
Male, %	89.5%	100%	66.7%	94.1%	0.034*
Age, mean (SD)	15.4 (2.6)	15.2 (2.8)	14.4 (1.7)	16.0 (2.9)	0.350
Weight, mean (SD)	57.7 (11.8)	56.6 (10.8)	53.9 (9.6)	60.4 (13.4)	0.395
LOS, median [IQR]	2.3 [0.7, 4.3]	4.4 [3.9, 5.3]	2.6 [2.3, 3.3]	0.7 [0.6, 1.3]	< 0.001
LOS > = 3 days, %	39.5%	100%	33.3%	0.0%	< 0.001

^€^Significance of between group differences in distribution for gender assessed using Fisher’s exact test used due to cell value < 5 patients, age and weight (ANOVA), and LOS (Kruskal–Wallis test)

*p < 0.05, ~ *p* >  = 0.05–< 0.10

The primary outcome of the study was LOS after the Nuss procedure, with focus on same-day discharge. Secondary outcomes included narcotic usage, post-operative pain, and the incidence of peri-operative complications. Specific data collected postoperatively included operative details, type and duration of patient analgesia administered, postoperative complications, LOS, pain scores, and total inpatient narcotic use. In order to streamline the comparison of narcotics used, all medications were converted into oral morphine equivalent (OME) in milligrams per kilogram [24].

### Patient selection and enrollment

All study participants met the following inclusion criteria: (1) diagnosis of pectus excavatum; (2) scheduled to undergo the Nuss procedure; (3) 12–30 years of age. Group 1 comprised patients retrospectively identified as having received bilateral PVBc, who served as historical controls. Group 2 patients received bilateral PVBc and right-sided cryoablation only. Group 3 patients received single-shot PVB and bilateral cryoablation. All patients in Group 3 were scheduled as first case of the day, which allowed the day for recovery and discharge in the evening of surgery. No patients were excluded.

### Nuss procedure and analgesia

A single pediatric surgeon performed all Nuss procedures (including groups 1, 2, and 3)with the assistance of a second attending pediatric surgeon. The Nuss procedure was performed for all patients as follows: the deepest part of the pectus deformity in the midline and the planned bar insertion sites in the intercostal spaces at the edges of the defect were marked. A bar appropriate for the size of the patient’s thorax was selected and bent to the appropriate shape. A lateral thoracic incision was made on each side of the chest wall in a transverse manner, just inferior to the level of planned bar placement, using cautery dissection down to the muscular fascia. Blunt dissection of the anterior chest wall to the medial border of the anterior pectus ridge was then performed. Using the lateral incision, entry was made into the chest with hemostat, and a 5-mm trocar was inserted. The thorax was insufflated to 5 mmHg using CO2, and thoracoscopy was performed. After dissection between the heart and the posterior sternum, and passage of Fiberwire (Arthrex, Naples, FL) using the passer, the bar was introduced with convexity facing posteriorly. The bar was then flipped so that the convexity was anterior inside the patient, which forced the sternum anteriorly for correction of the pectus excavatum. The bar was secured to the muscular fascia with a single stabilizer. The operating surgeon determined the location of fixation, the number of bars to be implanted (1–2), and the number of stabilizers to be used on a case-by-case basis.

For all patients, anesthetic management included efforts to prevent or reduce perioperative pain and nausea. As part of our protocol, starting 3 days before the procedure, Group 3 patients received daily treatment with 17 g polyethylene glycol and oral gabapentin 100 mg. The night before surgery, a single dose of diazepam 5 mg was administered orally to aid in anxiolysis. A transdermal scopolamine patch was placed preoperatively on the day of surgery to help control postoperative nausea. No patients in Groups 1 or 2 received preoperative medications from this protocol.

Intraoperative pain management for all groups included the induction of general anesthesia with intravenous propofol, rocuronium, and fentanyl (1–2 mcg/kg). Following induction, a double-lumen endotracheal tube was placed, with positioning confirmed by fiberoptic bronchoscopy. After the endotracheal tube had been secured Groups 1 and 2 had bilateral PVB and paravertebral catheters placed using an 18G Stimuplex Tuohy needle (B Braun, Bethlehem, PA). After ultrasound localization, catheters were threaded approximately 2–3 cm into paravertebral space and secured with Tegaderm (3 M, St. Paul, MN) for postoperative continuous infusion. Bupivacaine 0.125% was used to achieve a cumulative infusion rate of 0.3–0.4 mg/kg/hr. We hope to use liposomal bupivacaine in the future given its longer duration of effect. Group 3 patients received single-shot PVB, also using ultrasound guidance (Image 1) with maximum local anesthetic dose calculated for each patient based on weight, and a target volume of 20 mL 0.25% or 0.5% bupivacaine with epinephrine was given on each side (Image 2). Maintenance of general anesthesia was achieved with inhalational sevoflurane or desflurane. Antiemetic efforts included preoperative scopolamine patch (Group 3 only), intraoperative dexamethasone and ondansetron. If needed, 1–2 doses of intravenous hydromorphone hydrochloride 0.2 mg were titrated during bar placement. Ketorolac 0.5 mg/kg and intravenous acetaminophen 15 mg/kg were given prior to extubation.

For patients in Groups 2 and 3 (right-side or bilateral cryoablation), a separate dissection through the same lateral incision was performed to pass the cryoablation probe into the thorax over a rib. Cryoablation was performed on bilateral intercostal nerves (T3–T7) under thoracoscopic visualization. The tip of the cryoSPHERE probe (Atricure, Mason, OH) was allowed to reach − 69 °C for 1 cycle (2 min), and care was taken to ensure that the cooled probe surface did not touch the lung. In patients who received bilateral cryoablation, left thoracic cryoablation was performed first so that the pectus bar could be passed without having to deflate the right lung twice. After the procedure had been completed and the bar and stabilizer had been secured, capnothorax was evacuated by passage of a silicone stiff catheter into the chest under water seal and Valsalva inflation.

Chest X-ray was performed prior to closure of the skin to confirm adequate evacuation of capnothorax and lung inflation. No Foley catheter, arterial line, or chest tube was placed. Patients were extubated in the operating room.

### Postoperative care

Postoperatively all patients were taken to the post-anesthesia care unit (PACU) where intravenous hydromorphone hydrochloride and ondansetron hydrochloride were provided as needed for breakthrough pain or nausea. After recovery in the PACU, patients were admitted to the surgical floor. Post-operative pain was controlled orally and intravenously with non-narcotic and/or opioid medications.

All patients were advanced to a regular diet as tolerated and once fully awake after surgery, required to ambulate with assistance from physical therapists. Patients’ pain was assessed by the bedside nurse every 4 h. Patients in Group 3 received scheduled Tylenol and Toradol; oral acetaminophen-hydrocodone and intravenous hydromorphone hydrochloride were given as needed. Patients in Groups 1 and 2 received a mixed combination of Tylenol, Toradol, oral hydrocodone, oral oxycodone, intravenous morphine, and patient-controlled analgesia. Notably, oral hydrocodone and oral oxycodone are roughly 1.5 times more potent than oral morphine; parenteral morphine is 3 times as potent as oral morphine and parenteral hydromorphone is almost 20 times as potent as oral morphine [24]. All narcotic doses were converted to oral morphine equivalent (OME) doses in mg OME/kg using the following recommended formula from Neilson et al.: strength per unit × (number of units/day (or total)) × OME conversion factor = OME units per day (or total) [24].

For patients in Group 3, discharge readiness was determined by the pediatric surgery nurse practitioner based on patient reported tolerable pain control with oral medications, ability to walk independently, diet tolerance, and family comfort with same-day discharge. Patients in Groups 1 and 2 were not hospitalized on a study protocol and, therefore, did not receive pre- or post-operative protocolized medications, or assessment for same-day discharge.

### Follow-up

Upon discharge, all patients were sent home with pain medications (narcotic and non-steroidal anti-inflammatory/acetaminophen) and Group 3 patients were instructed to keep a pain and medication log (supplemental Table 1). There was very limited information found via chart review about Groups 1 and 2 regarding pain score or specific medications taken once they were discharged. Patients were followed at 3 weeks, 3 months, 12 months, and 18 months after surgery. Each follow-up visit consisted of an interim history including pain scores, and a focused physical exam, including specific assessment of incision sites, and chest wall sensation on patients receiving cryoablation. The bar implanted during the Nuss procedure was removed at 2 years after surgery and at 3 years if the patient was older than 20 years or had confirmed or highly suspected connective tissue disease.

### Endpoints and data analysis

Characteristics of the study population were described as percentage with defined trait for categorical variables, and as mean [standard deviation (SD)] or median [interquartile range (IQR)] for continuous variables. The primary outcome in this study was postoperative LOS, which is an objective measure that synthesizes numerous aspects of a patient’s postoperative course, including adverse events andpain control.. Secondary outcomes included narcotic usage (mg OME/kg), pain scores, side effects related to use of paravertebral regional anesthesia or cryoanalgesia and procedural complications.

Differences between treatment groups in gender distribution and use of narcotic medication were assessed for significance with Fisher’s exact test. Analysis of variance (ANOVA) was performed to identify group differences in age and weight; differences in LOS were assessed with the Kruskal–Wallis test, with two-group comparisons based on the Mann–Whitney *U*-test. All analyses were conducted using SPSS V (SPSS Inc, Chicago, IL).

## Results

In patients who underwent the Nuss procedure for the treatment of pectus excavatum, 12 had no cryoablation (Group 1), 9 had right-side cryoablation (Group 2), and 17 had bilateral cryoablation (Group 3). No patients in Group 3 required hospital LOS >  = 3 days (Table 1). One-third of those who had right-side cryoablation (Group 2) and all patients with no cryoablation (Group 1) required LOS >  = 3 days, *p* < 0.001. No patients in Groups 1 and 3 required hospital LOS >  = 3 days compared to a third of patients in Group 2, *p* = 0.010.

### Primary outcome

**Fig. 1:**
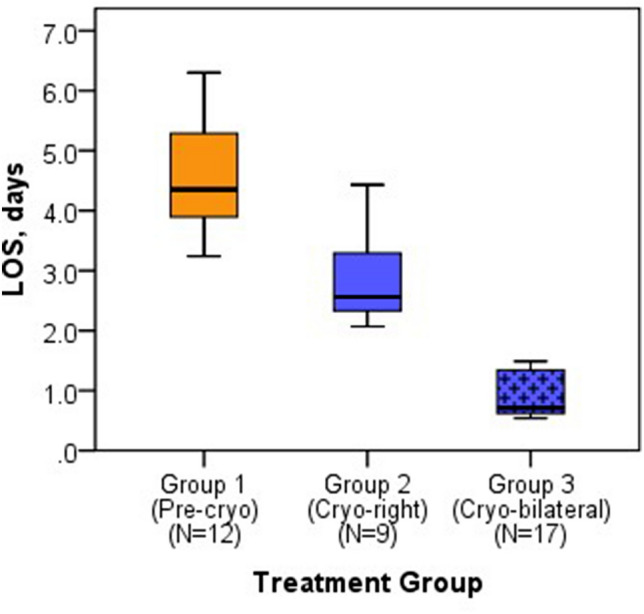
LOS distribution described by treatment group: LOS overall median [IQR] 2.3 [0.7, 4.3], Group 3 median IQR 0.7 [0.6, 1.3]. LOS >  = 3 days: Group 1 median IQR 100%; Group 2 median IQR 33.3%; Group 3 median IQR 0.0% (*p* < 0.001)

Patients who had bilateral cryoablation (Group 3) had significantly reduced average LOS (median = 0.7 days IQR: 0.6, 1.3) compared to those who had right-side cryoablation (median = 2.6 days [IQR: 2.3, 3.3] and no cryoablation (median = 4.4 days [IQR: 3.9, 5.3], *p* < 0.001 (Table 1, Fig. 1). The shorter average LOS seen in patients who had bilateral cryoablation was significant when compared to both the groups who had right-side cryoablation (Group 2) and no- cryoablation (Group 1) (*p* < 0.001). Nearly 65% (11 of 17) of patients in the bilateral cryoablation group were discharged home on the day of surgery compared to no patients in the other procedure groups. No patients were readmitted.

### Secondary outcomes

#### Analgesia requirements

**Fig. 2:**
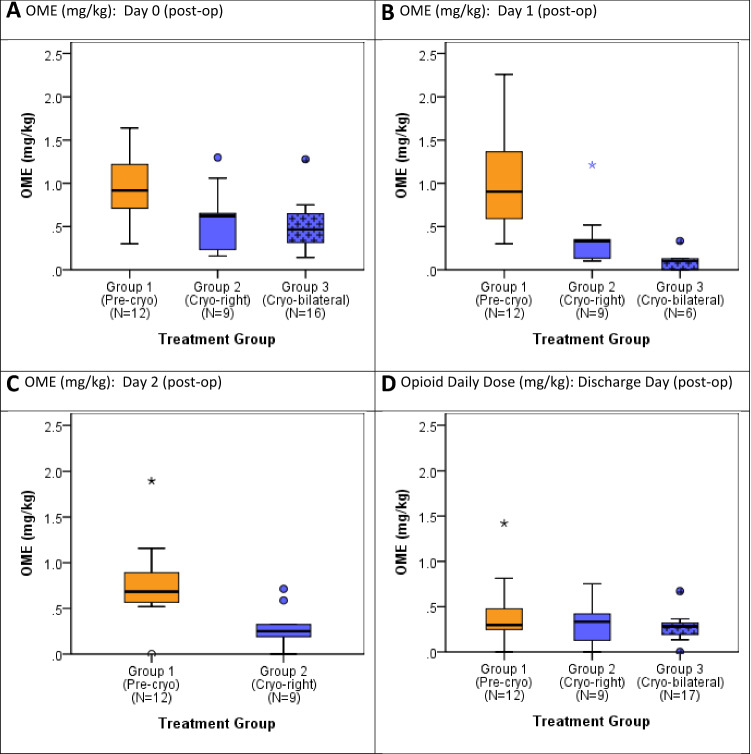
Oral morphine equivalent (OME, mg/kg) daily dose distribution described for each group post-op days 0, 1, 2 and at discharge (* indicates one extreme outlier on day 1 not presented on chart (OME on day 1 = 3.11 mg/kg, beyond Q3 + 1.5 * IQR)

As previously noted all administered opioid medications were converted to daily OME doses in milligrams per kilogram for ease of comparison [24]. The opioid usage for each treatment group is presented in Fig. 2. Patients who underwent bilateral cryoablation required significantly less opioid analgesia in OME in mg/kg doses than those who did not receive cryoablation. Using this single metric, we showed that Group 1 (no cryoablation) used nearly two times the OME doses of narcotics used by Group 3 (0.92 and 0.47 mg/kg, respectively, *p* = 0.10) on the day of surgery (postoperative day 0, in Table 2). By postoperative day 2, all patients from Group 3 had been discharged. On postoperative day 3, opioid requirement was similar between Groups 1 and 2 (*p* = 0.072). Notably, Group 3’s total opioid use was significantly decreased compared to other groups throughout their hospitalization, while Groups 1 and 2 had high opioid use on postoperative days 1–3. Group 3 had adequate pain control on oral medications and minimal reported opioid use after discharge (supplemental Table 1).

**Table 2:** Daily average oral morphine equivalent medication dosage in milligrams per kg (OME, mg/kg) compared across groups

Postoperative day	Group 1 (Pre-cryo)		Group 2 (Cryo-right)		Group 3 (Cryo-bilateral)		Test of group differences^a^			
							Overall	1 vs. 2	1 vs. 3	2 vs. 3
	*N*^b^	Median [IQR] OME, mg/kg	*N*	Median [IQR]	*N*	Median [IQR]	*P* value	*P*-value	*P*-value	*P*-value
Day 0	12	0.92 [0.71,1.22]	9	0.62 [0.23, 0.65]	17	0.47 [0.31, 0.65]	0.004*	0.023*	0.001*	0.821
Day 1	12	0.91 [0.59, 1.36]	9	0.33 [0.13, 0.35]	6	0.10 [0.00, 0.13]	< 0.001*	0.004*	0.001*	0.013*
Day 2	12	0.68 [0.57, 0.89]	9	0.25 [0.19, 0.32]	0	–	–	0.006*	–	–
Day 3	12	0.30 [0.25, 0.48]	4	0.34 [0.29, 0.52]	0	–	–	0.544	–	–
Day of discharge	12	0.30 [0.25, 0.48]	9	0.28 [0.19, 0.32]	17	0.33 [0.13, 0.42]	0.702	0.414	0.595	0.686

^a^Significance of distributional differences between groups assessed by Kruskal–Wallis test overall and Mann–Whitney *U* test for two group comparisons

^b^N indicates number of patients requiring opioid medication on respective day

*p < 0.05

Limited data to test for significance of difference

IQR = Interquartile range [25th, 75th percentiles]

#### Post-operative pain

**Fig. 3:**
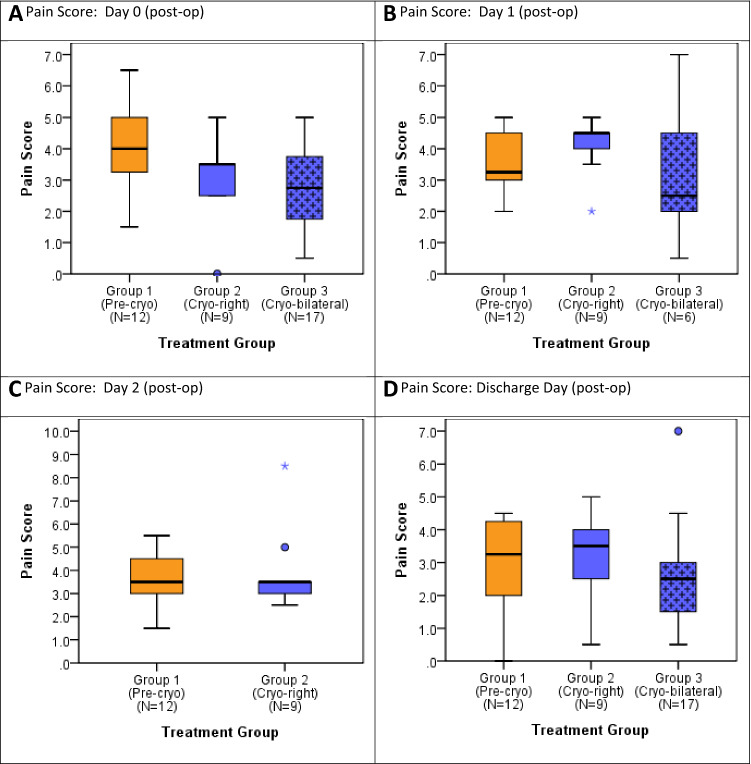
Pain score distribution described for each group post-op days 0, 1, 2 and at discharge

Post-operative pain scores were lower in patients who underwent cryoablation compared to those who did not. Daily average pain scores for all treatment groups are presented in tabulated format in Table 3 and in -and-whisker plots in Fig. 3. Pain scores on Day 0 were significantly lower in Group 2 than in Group 1 (*p* = 0.034) (Table 3; Fig. 3A) and lower in Group 3 than in the other two groups; however, the latter trend did not reach significance. On Day 1, pain scores were highest in Group 2, followed by Group 1, and then by Group 3 (most patients were discharged on POD 0, remaining patients *N* = 6), although the trend was not significant (Fig. 3). By POD 2, pain scores were similar in Groups 1 and 2, and pain had resolved sufficiently for discharge from the hospital for all patients from Group 3. On Day 3, pain scores were slightly lower in Group 2, compared with Group 1. When pain scores on the day of discharge were compared among groups, pain scores were lower in the patients who underwent bilateral cryoablation, compared to patients who received no cryoablation or right-side cryoablation only, but this trend did not achieve significance.

**Table 3:** Daily average pain score compared across groups. *Note:* One patient in cryo-bilateral group had first measurements on day 1 (reason day 0 = 16 patients)

Postoperative day	Group 1 (Pre-cryo)		Group 2 (Cryo-right)		Group 3 (Cryo-bilateral)		Test of group differences^a^			
							Overall	1 vs. 2	1 vs. 3	2 vs. 3
	N	Median [IQR]	N	Median [IQR]	N	Median [IQR]	*P*-value	*P*-value	*P*-value	*P*-value
Day 0	12	4.0 [3.3, 5.0]	9	3.5 [2.5, 3.5]	16	2.8 [1.8, 3.8]	0.078	0.170	0.026*	0.548
Day 1	12	3.3 [3.0, 4.5]	9	4.5 [4.0, 4.5]	6	2.5 [2.0, 4.5]	0.307	0.294	0.317	0.209
Day 2	12	3.5 [3.0, 4.5]	9	3.5 [3.0, 3.5]	0	–	–	0.665	–	–
Day 3	12	3.3 [2.0, 4.3]	4	3.0 [1.3, 4.3]	0	–	–	0.854	–	–
Day of dscharge	12	3.3 [2.0, 4.3]	9	3.5 [2.5, 4.0]	17	2.5 [1.5, 3.0]	0.343	0.802	0.284	0.182

^a^Significance of distributional differences between groups assessed by Kruskal–Wallis test overall and Mann–Whitney *U* test for two group comparisons

*p < 0.05

Limited data to test for significance of difference

IQR = Interquartile range [25th, 75th percentiles]

#### Operative outcomes

All patients had successful correction of pectus excavatum. Among Group 3 patients who were not discharged on the day of surgery, one patient had initial urinary retention. The patient’s symptoms had resolved by the evening of Day 0 and required no further intervention over follow-up. There was also one occurrence of Horner’s syndrome from PVBC placement with spontaneous resolution after discontinuation of the infusion. One patient developed a bar infection that resolved without removal and no incidence of bar displacement. There were no clinically significant pneumothoraces/capnothoraces that required treatment. The protocol used in this study added approximately 30 min to time in the operating room.

## Discussion

Many approaches to multimodal pain management have been proposed over the years to establish and improve ERAS specific to the Nuss procedure [14]. Over time, protocols at our institution for pain control after the Nuss procedure progressed from use of epidural and patient controlled analgesia (medication driven) to a multi-modal approach which was more procedurally based. We believe the specific combination of PVB and bilateral thoracic intercostal nerve cryoablation decreased LOS and significantly impacted pain control as opposed to either isolated PVB or isolated bilateral thoracic intercostal nerve cryoablation. In our experience this is due to the fact that PVB, similar to intercostal nerve blocks, provide immediate pain control to bridge the gap of time (up to 12 h) before pain control from cryoablation takes effect. This is why studies reporting on intercostal nerve cryoablation have never reported same day discharge. PVB with experienced anesthesiologists familiar with this procedure are able to achieve this as expeditiously (less than 10 min) as individual intercostal nerve blocks and provide a broader distribution with fewer injection sites needed. In group one we identified patients receiving bilateral PVB with placement of PVB-catheter (PVBc) for continuous analgesic infusion and no cryoablation. Next, in group 2, we transitioned to the use of bilateral PVBc in combination with only single (right)-side thoracic intercostal nerve cryoablation, due to initial lack of data on nerve regeneration and return of sensation to the chest with this technique. However, it soon became evident based on experience at our institution, as well as findings reported in the literature, that bilateral cryoablation was extremely safe and effective [19]. We also observed that LOS and postoperative opioid requirements were reduced in patients treated with unilateral cryoablation and bilateral PVBc placement. Thus, we formulated an approach including bilateral PVB with bilateral cryoablation (group 3) to assess the feasibility of comprehensive short and long term pain management allowing for earlier discharge.

We then conducted a pilot study to compare the effect of this multi-modal perioperative pain management with bilateral single-shot PVB and bilateral thoracic intercostal nerve cryoablation, compared to our previous approaches of the following: (1) bilateral PVB with in-dwelling catheter infusion, and (2) bilateral PVB with in-dwelling catheter infusion along with right-sided thoracic intercostal nerve cryoablation. Our results showed shorter LOS and decreased postoperative opioid requirement in patients who received bilateral intercostal cryoablation and bilateral single-shot PVB, compared to the approaches used previously, with no readmissions. This is the first study to show that combined pain block treatment with bilateral PVB and bilateral cryoablation allows for same-day discharge after the Nuss procedure for repair of pectus excavatum.

The results of our comparative study support other published reports of significant decreases in LOS and opioid usage in patients treated with cryoablation for pain management after the Nuss procedure [17, 18]. However, the effects of intercostal nerve cryoablation are delayed, which shifts the need for postoperative pain control to an earlier time period [18, 25]. At our institution, we recently implemented a peri-operative multimodal pain management plan that includes single-shot PVB without catheter placement and bilateral thoracic intercostal nerve cryoablation. In this pilot study, we investigated the effectiveness of this pain management protocol for alleviating acute pain from the procedure along with chronic pain from stretching of the bony structures in the chest after Nuss bar placement.

In a comparison of paravertebral to epidural blocks, Aydin et al. reported that patients had better pain management, decreased LOS, and increased satisfaction if they were administered either PVB or epidural block, compared to neither, prior to surgery [26]. However, the placement of epidural block or catheter carries risk for nerve injury. Paravertebral blocks with or without continuous infusion catheter for pain management after repair of pectus excavatum have not led to permanent nerve injury at our institution. We have had one case of Horner’s syndrome that resolved after discontinuation of the infusion. The study by Aydin et al. showed that both groups had lower LOS than patients who received only intravenous medications, with no significant difference between paravertebral vs. epidural block. However, comparisons of epidural blocks with cryoablation have demonstrated significant differences in pain relief, LOS, total opioid usage, and duration of opioid usage [18, 25].

 Hypotensive episodes can occur with high paravertebral catheter placement infusions and are reported in the literature; the use of clonidine patches could simultaneously aid in pain control as well as decrease the risk of hypotension [27]. In this series, no intra-operative hypotensive episode requiring any intervention was observed, demonstrating the safety of the multimodal pain bundle. We have not observed any hypotensive episodes, and do not use clonidine patches as part of our current protocol although they may have utility in the future.

While this study presents pilot feasibility data that multimodal pain management protocols can provide improved pain control and, in some patients, allow for same-day discharge, this should in no way be taken as evidence that all patients should be discharged on the same day. The optimization of a same-day ERAS protocol is multifactorial. In this study, reasons unrelated to pain for why some patients did not meet same-day discharge criteria included anxiety about going home, urinary retention. No patient returned for readmission, and no missed or delayed complications specific to the multimodal pain bundle were identified during outpatient follow-up.

The substantial improvements in pain management, with the possibility of same-day discharge, and substantially decreased opioid usage reported here are critical to optimizing the core component of ERAS guidelines. Rettig et al. recently investigated the effect of intraoperative intercostal nerve block injection on same-day discharge after the Nuss procedure [28, 29]. They reported same-day discharge without subsequent complications in 10 of 15 patients, which is similar to the rate observed in our study population. Furthermore, multimodal pain management after the Nuss procedure benefits not only the patient but also the hospital. Benefits include early discharge in the context of the global COVID-19 pandemic, avoiding increase in risk of hospital-acquired infection, as well as optimal space utilization within hospitals. These findings also demonstrate the need for effective acute pain management using intercostal nerve block or PVB placement in combination with bilateral intercostal nerve cryoablation for chronic pain control. Intercostal nerve block and PVB placement are equally effective, but the latter requires a high degree of anesthesia skill for accurate placement. Clinical direction will be key to safe approaches to same-day discharge, and patient comfort and safety should always remain paramount in the decision process.

We would like to note that there are multiple limitations to this study. This is a pilot study reflecting our evolving experience. The findings presented above may lay the foundation for a larger multi-institutional study. While our results showed that it is feasible to discharge Nuss procedure patients on the day of surgery, this finding should be interpreted with caution, and not all patients will be eligible for early discharge. We acknowledge that this multimodal approach, and not the cryoablation alone, contributed to same day discharge. Patients were specifically scheduled as first case operations to allow adequate time during the day for the patient to recover, achieve pain control with oral medications, be seen by physical therapy and be able to spontaneously void. The criteria to meet same day discharge required at least 6 h and did not allow us to consider this in patients undergoing operations later in the day as part of our pilot study. This was a focused effort to achieve same day discharge which was not considered on the previous group of patients.The limitations of this study also include the lack of analysis of additional costs related to use of the cryoablation. As noted above, cryoablation added approximately 30 min of operating room time. Future research should include a cost–benefit analysis of the proposed protocol as well as a comparison of intercostal vs. paravertebral nerve block in terms of patient outcomes. A multi-site study with a larger sample size may elucidate the effects of multimodal pain management after the Nuss procedure on patient satisfaction as well as the cost savings associated with decreased LOS.

The results of this pilot study demonstrate that combining intraoperative bilateral intercostal nerve cryoablation with PVB shortens LOS and reduces opioid use after the Nuss procedure, compared with PVBc alone or with PVBc plus right-side cryoablation. The combination of bilateral cryoablation and PVB provided superior pain control, compared with the pain management approach used for historical cohorts of Nuss procedure patients at our institution. Finally, this improved multi-modal analgesic approach does not appear to be associated with increased risk of complications, as there were no readmissions.

### Supplementary Information

Below is the link to the electronic supplementary material.

Supplementary file1 (DOCX 223 KB)

Supplementary file2 (DOCX 774 KB)

Supplementary file3 (DOCX 16 KB)

## Data Availability

All data generated or analyzed pertinent to the study are included in this published article.

## Abbreviations

ANOVA: Analysis of variance
ERAS: Enhanced recovery after surgery
IQR: Interquartile range
LOS: Length of stay
MIRPE: Minimally invasive repair of pectus excavatum
PACU: Post-anesthesia care unit
PVB: Paravertebral block
PVBc: Paravertebral block with continuous infusion catheter
SD: Standard deviation
