# Supplementary material for: A pilot study of multi-modal pain management for same-day discharge after minimally invasive repair of pectus excavatum (Nuss procedure) in children
Source: Pediatr Surg Int. 2023 Mar 26;39(1):159. doi: 10.1007/s00383-023-05429-7 (PMC10040230; doi:10.1007/s00383-023-05429-7)

**Image 2. Injection of Bupivacaine for paravertebral block in the extra pleural space as shown by bulging of the pleural lining downward and blue arrow**


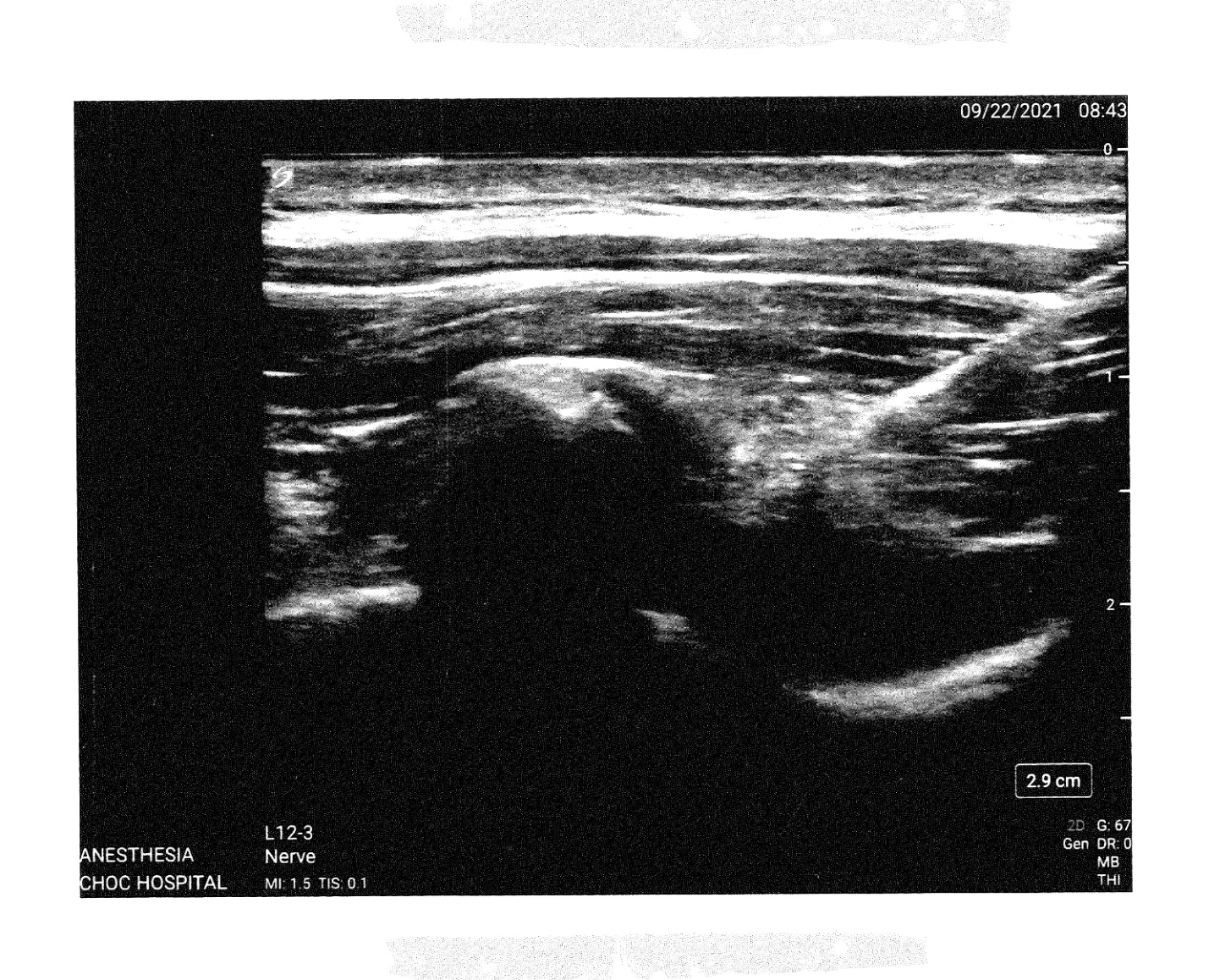

Supplement: Supplementary file 2 — Supplementary file2 (DOCX 774 KB) [file 383_2023_5429_MOESM2_ESM.docx]
