# Supplementary material for: A pilot study of multi-modal pain management for same-day discharge after minimally invasive repair of pectus excavatum (Nuss procedure) in children
Source: Pediatr Surg Int. 2023 Mar 26;39(1):159. doi: 10.1007/s00383-023-05429-7 (PMC10040230; doi:10.1007/s00383-023-05429-7)
Supplement: Supplementary file 3 — Supplementary file3 (DOCX 16 KB) [file 383_2023_5429_MOESM3_ESM.docx]

**Supplemental Table 1.** Qualitative responses (if provided) from Group 3 patients at post-op 3-week and 3-month follow-up(s)

|  | | | Reported pain score* | Describe pain | Prn pain meds |
| --- | --- | --- | --- | --- | --- |
| Group 3 (cryo - bilateral) | Pt1 | ~3 weeks post-op | 2 | occasional sharp chest pain | Tylenol/Motrin every 4hours and took a total of twenty tablets in first week. |
|  |  | ~3 months post-op | 1 |  | no pain medications, |
|  | Pt2 | ~3 months post-op | 2 | pain outside incision | did need Acetaminophen-hydrocodone 5-325 for up to 2 weeks. |
|  | Pt3 | ~3 months post-op | 0 |  | 3 Acetaminophen-hydrocodone 5-325 tablets on pod 1 and only Tylenol or Motrin |
|  |  | ~3 weeks post-op | 6 | some heaviness or numbness in mid chest, no tingling | no medications now |
|  | Pt4 | ~3 weeks post-op | 0 | a lot better, feels like normal. no pain outside incision | no Acetaminophen-hydrocodone 5-325 and only one tablet of Tylenol |
|  | Pt5 | ~3 months post-op | 4 | numb on right side, some sensation returning on left |  |
|  |  | ~3 weeks post-op | 2 | starting to return from left side, mid to lateral right side just starting | 1 acetaminophen-hydrocodone 5-325 per day x1 week |
|  | Pt6 | ~3 weeks post-op | 10 | pressure, 1.5 weeks after surgery was having pain 10, currently (at time of appt), 2-6 | 2 acetaminophen-hydrocodone 5-325 per day x3 weeks |
|  | Pt7 | ~3 weeks post-op | 4 | burning on right side anterior to incision | Tylenol once per day, acetaminophen-hydrocodone 5-325 q6 x1 wk |
|  | Pt8 | ~3 weeks post-op | 7 | mid chest in the am and when sneezing |  |
|  |  | ~3 months post-op | 1 | since surgery pain is about 1, first wk maybe a 7 |  |
|  | Pt9 | ~3 months post-op | 8 | occasional soreness in back | Aleve |
|  |  |  |  |  |  |

*Pain reported on a 0-10 scale where 0=no pain, and 10= worst pain
